# Non-Immunotherapy Application of LNP-mRNA: Maximizing Efficacy and Safety

**DOI:** 10.3390/biomedicines9050530

**Published:** 2021-05-10

**Authors:** Irena Vlatkovic

**Affiliations:** BioNTech SE, 55131 Mainz, Germany; irena.vlatkovic@biontech.de

**Keywords:** lipid nanoparticle, LNP-mRNA, innate immunity, efficacy, safety, cytokines, non-immunotherapy applications, RNA protein replacement therapy, rare disease, in vitro transcription (IVT)

## Abstract

Lipid nanoparticle (LNP) formulated messenger RNA-based (LNP-mRNA) vaccines came into the spotlight as the first vaccines against SARS-CoV-2 virus to be applied worldwide. Long-known benefits of mRNA-based technologies consisting of relatively simple and fast engineering of mRNA encoding for antigens and proteins of interest, no genomic integration, and fast and efficient manufacturing process compared with other biologics have been verified, thus establishing a basis for a broad range of applications. The intrinsic immunogenicity of LNP formulated in vitro transcribed (IVT) mRNA is beneficial to the LNP-mRNA vaccines. However, avoiding immune activation is critical for therapeutic applications of LNP-mRNA for protein replacement where targeted mRNA expression and repetitive administration of high doses for a lifetime are required. This review summarizes our current understanding of immune activation induced by mRNA, IVT byproducts, and LNP. It gives a comprehensive overview of the present status of preclinical and clinical studies in which LNP-mRNA is used for protein replacement and treatment of rare diseases with an emphasis on safety. Moreover, the review outlines innovations and strategies to advance pharmacology and safety of LNP-mRNA for non-immunotherapy applications.

## 1. Introduction

The host immune system recognizes and responds to viral infections. Key responders of the innate immune system in anti-viral defense are pattern recognition receptors (PRRs) that target viral genomic DNA and RNA, such as single-stranded RNA (ssRNA) and double-stranded RNA (dsRNA). Activation of PRRs leads to signal transduction cascades resulting in secretion of cytokines and development of adaptive immunity. Similar to viral nucleic acids, messenger (m)RNA-based vaccines and therapeutics activate the immune system through the same mechanisms based on PRR recognition, reviewed in [1,2,3,4]. They represent a novel class of drugs and consist of synthetic mRNA packed into diverse types of shields that protect the mRNA from ribonucleases (RNases) and facilitate the transport and introduction of mRNA into the target cells, tissues, and organs. In vitro transcribed (IVT) mRNA-encoded antigens (for vaccine and immunotherapy applications) or the protein of interest (for therapeutics as protein replacement therapies or antibody production) is commonly encapsulated into lipid nanoparticles (LNP). In protein replacement therapies, mRNA is engineered to code for an intracellular or secreted protein of interest [1,5]. In typical cases, protein replacement aims to restore enzyme function to treat rare monogenic diseases. When LNP-encapsulated mRNA enters the cytoplasm, the cellular translational machinery reads the protein of interest from LNP-delivered mRNA matrices. The therapeutic protein is modified post-translationally in a host-cell specific manner, which is one of the major advantages compared with enzyme replacing therapies (ERT) that directly utilize protein administration [5]. Other advantages of mRNA over protein for protein replacement therapies are overcoming challenges in production and degradation of large biomolecules, as well as difficulties in the delivery of intracellular and transmembrane proteins in ERTs [6]. mRNA therapeutics are characterized by a relatively fast, simple, and inexpensive production [1]. When compared with other nucleic-acid-based therapies (e.g., DNA-based vaccines), mRNA-based therapeutics have several advantages: lack of genomic integration, functionality in cytoplasm, and no requirement for nuclear targeting [1,5]. Preclinical studies examining the basis of mRNA technology started already 30 years ago, with the first demonstration by Wolff et al. showing that naked IVT mRNA injected into mice can be translated [7]. Recently, a number of LNP-mRNA vaccines for infectious diseases, mRNA-based cancer immunotherapies, and several RNA protein replacement therapeutics entered clinical trials [5,8]. In December 2020, LNP-mRNA vaccines against SARS-CoV-2 from BioNTech/Pfizer and Moderna were demonstrated to be highly effective and safe in a Phase 3 clinical trial in preventing symptomatic COVID-19, thereby obtaining emergency use authorizations or conditional marketing authorizations in several countries worldwide, giving hope for an end of the COVID-19 pandemic [9,10,11]. Recently, LNP-mRNA from CureVac also entered Phase 3 clinical trial, increasing the variety of LNP-mRNA pipelines against COVID-19 [12]. Background information from pivotal primary studies and experiences from the previous Phase 1 and Phase 2 clinical trials using mRNA-based technology were the basis for the quick design and production of large amounts of an efficient and safe LNP-formulated mRNA-based vaccine in the COVID-19 pandemic, paving the way to a promising future of this field [13,14]. LNP-mRNA prophylactic vaccines and immunotherapy applications can overall benefit from adjuvant effects on immune activation through PRRs [15,16]. However, LNP-mRNA-based non-immunotherapy applications typically require long-term repetitive systemic administration, cell-specific targeting of mRNA, high translation of the target protein, and maximal safety without immune activation. These application types, such as protein replacement therapies, need further understanding of the mode of action and further maximization of their efficacy and safety. Thus, while many preclinical studies are currently ongoing, there are only a few mRNA-based non-immunotherapy drug candidates that have entered clinical studies. This review summarizes the basics of IVT mRNA, byproducts and LNP-induced immune activation, as well as the current state of RNA-based non-immunotherapy applications. The review further analyzes the current solutions for boosting efficacy, safety, and future considerations for developing LNP-mRNA therapeutics.

## 2. IVT mRNA and Byproducts Induced Immune Activation

The mRNA component of LNP-mRNA-based therapeutics is produced synthetically during in vitro transcription reaction using cap, ribonucleotides, and DNA template containing a promoter, as well as a phage RNA polymerase recognizing that promoter. In vitro transcribed mRNA typically consists of a cap structure, 5′ untranslated region (5′UTR), codon optimized protein coding or antigen sequence, 3′UTR, and polyA tail [1]. IVT mRNA is designed to resemble natural mRNAs while engineering maximal possible benefits in translational properties and pharmacodynamics, as well as mRNA stability and safety. However, the IVT reaction components and conditions can lead to a production of not only the mRNA of interest, but also of diverse amounts of immunostimulatory byproducts/contaminants such as dsRNA [17,18,19]. In addition, one must ensure that contaminant levels such as lipopolysaccharides (LPS)/endotoxin are absent or below well-established safety thresholds.

**Figure 1:**
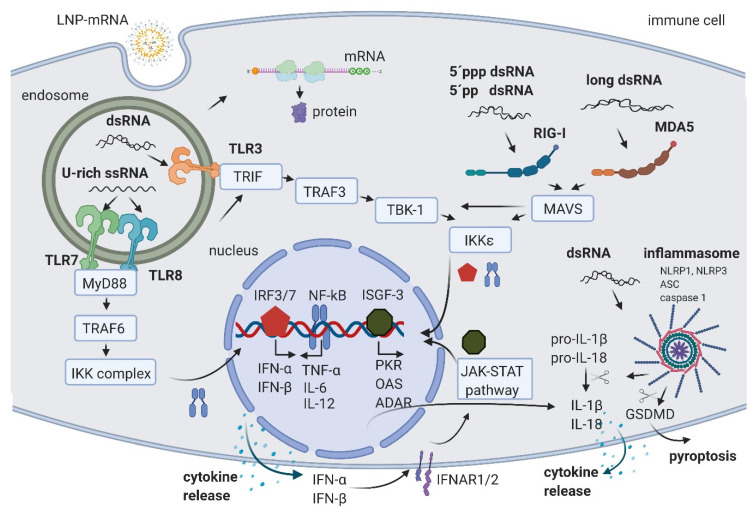
Schematic of RNA-induced immune activation. LNP-mRNA is endocytosed by cell-specific mechanisms. Small fractions of mRNA and byproducts of IVT reaction are released to cytoplasm by endosomal escape. In the cytoplasm, mRNA is translated by cellular machinery. In endosomes, U-rich ssRNA is sensed by TLR7 and TLR8, and dsRNA is detected by TLR3. The activation signal is transferred through the signal transduction cascade to the nucleus where the transcription factors NF-kB, IRF3, and IRF7 promote the production of cytokines. The released IFN-α and IFN-β are recognized by their receptor IFNAR, leading to the activation of the JAK-STAT pathway and the formation of ISGF-3, which activates the transcription of multiple hundreds of genes including PKR, OAS, and ADAR. In the cytoplasm, 5′ppp dsRNA and 5′pp dsRNA are recognized by RIG-I, and long dsRNA by MDA5. Both pathways lead to the additional increase in NF-kB, IRF3, and IRF7. Moreover, dsRNA can also activate the inflammasome through NLRP1 or NLRP3, leading to the cleavage of pro-IL-1β and pro-IL-18 by caspase 1 and IL-1β and IL-18 release, or a GSDMD cleavage followed by pyroptosis. Created with BioRender.com.

The level of immune activation by LNP-mRNA depends on the route of administration, dose, pre-existing immune state of model organism/patient, and on the features of the LNP-mRNA. The LNP-mRNA features to consider are: (1) mRNA modification/sequence/structure, (2) manufacturing of mRNA and IVT reaction byproducts/contaminants, and (3) features of the used LNP. The immune system can be activated by sensing RNA products and byproducts of IVT reaction by host PRRs. There are currently three main types of PRRs known. While Toll-like receptors (TLRs) mainly reside in the endosomal compartment of immune cells, Retinoic acid-inducible gene I (RIG-I)-like receptors (RLRs) and NOD-like receptors (NLRs) are found in the cytosol of immune and non-immune cells [2,20]. Sensing RNA by PRRs triggers signal transduction cascades leading to cytokine secretion and may finally result in immune system activation and in some cases even in cell death (Figure 1). Thus, RNA sensing by PRRs may lead to a decrease in the potency of LNP-mRNA and potential safety considerations, which is of particular interest for non-immunogenic LNP-mRNA applications. The following section summarizes the main immune activation pathways by giving an overview of the effects of uridine-rich (U-rich) single-stranded mRNA and diverse types of double-stranded RNA.

U-rich single-stranded mRNA is recognized by TLR7 and TLR8 [21,22]. Its recognition leads to TLR activation, signal transduction through myeloid differentiation primary response gene 88 (MyD88) adaptor protein, and tumor necrosis factor receptor associated factor 6 (TRAF 6), leading to the activation of IκB kinase (IKK) complex, reviewed in [2,23]. The IKK complex activates the NF-κB transcription factor (TF) that translocates to the nucleus where it induces the expression of type I interferons such as IFN-α and IFN-β, tumor necrosis factor-α (TNF-α), interleukin-6 (IL-6), and interleukin-12 (IL-12). This triggers the pro-inflammatory response and both autocrine and paracrine secretion of IFNs. IFNs then activate the Janus kinase (JAK)-signal transducer and activator of transcription (STAT) (JAK-STAT) pathway, leading to the formation of IFN stimulated gene factor 3 (ISGF-3), which in turn translocates to the nucleus, further activating hundreds of IFN-stimulated genes (the signaling cascades extensively reviewed in [2,23,24]). These genes include PRRs and TFs, further amplifying the signal and leading to a boost of the immune system activation.

In addition, ISGF-3 activates a number of genes having anti-viral/anti-RNA response functions, such as dsRNA-dependent protein kinase (PKR), 2′-5′-oligoadenylate synthetase (OAS), and RNA-specific adenosine deaminase (ADAR) (Figure 1) (reviewed in [2,20]). The activated PKR can phosphorylate eIF2α transcription initiation factor, leading to the inhibition of translation and stimulation of IKK complex, thereby amplifying the innate immunity signals or leading to apoptosis [25]. Double-stranded RNA-activated OAS synthesizes 2′-5′-linked oligoadenylates (2-5A) from ATP, which activate RNAse L, leading to cleavage and degradation of ssRNA [26]. Portions of mRNA cleaved by RNase L bind and activate PRRs, further amplifying the type I IFN loop.

An additional important mechanism of ISGF-3 gene activation includes an increase in Adenosine Deaminase acting on RNA (ADAR) enzymes. ADAR1 has multiple functions. First, ADAR1 targets double-stranded regions of mRNA molecules, deaminates adenosine (A) to inosine (I), thus introducing I:U mismatches, which leads to mRNA destabilization [27]. Introduced mismatches may lead to change in the amino acid sequence of the coded protein, which results in a lower translational efficiency of mRNA [28,29]. The second important function of ADAR1 is its role in the suppression of interferon signaling [30]. Liddicoat et al. generated mice with an editing-deficient knock in mutation [31]. Interestingly, embryonic death and phenotypes of these mice could be rescued when melanoma differentiation-associated protein 5 (MDA5) was concurrently deleted. In this study, the authors established ADAR1 physiological function in editing endogenous RNA and preventing its sensing as a nonself by MDA5, thus suppressing IFN response [31]. Moreover, another study reported that the lethal phenotype of ADAR1 deletion in human cells was rescued when RNase L was concurrently deleted [32]. This showed that ADAR1 also blocks the OAS-RNase L pathway [32,33]. While several in vitro studies suggested that ADAR1 also blocks RIG-I activation, this could not be confirmed in vivo, since editing-deficient mice could not be rescued with the concurrent deletion of RIG-I, requiring further studies (reviewed in [33]). In summary, an important role of ADARs in balancing immune activation and self-tolerance was established [31,33]. While U-rich ssRNA is sensed by TLR7 and TLR8, the dsRNA byproducts/contaminants are typically sensed in the endosomal compartment of macrophages by TLR3 [34,35] (Figure 1). TLR3 further activates TIR-domain-containing adapter-inducing interferon-β (TRIF) and TNF receptor associated factor 3 (TRAF3), TANK-binding kinase 1 (TBK1), and IKKε, respectively. This is followed by the activation of interferon regulatory factors (IRFs) 3 and 7, transcription factors that promote the production of type I IFNs, finally causing signal amplification and immune system activation through the already described pathways (reviewed in [2,23]).

In both immune and non-immune cells, dsRNA is recognized by RLRs and NLRs (reviewed in [20,36]). RIG-I is a RLR that recognize 5′ppp dsRNA and 5′pp dsRNA [37]. This recognition requires the base pairing of the nucleoside carrying 5′ppp and its lack of N1-2’O-methylation [38,39]. A long dsRNA (larger than 1kb) is recognized by MDA5 RLR [40,41]. In both cases of dsRNA sensing by RIG-I and MDA5, the signal is transferred to mitochondrial antiviral-signaling protein (MAVS) and leads to the activation of the TBK1 and IKKξ [42]. dsRNA can also be recognized by two members of the NOD-like receptors family (NLRs): activated NLR family pyrin domain containing 1 (NLRP1) or NLRP3, which, together with the apoptosis-associated speck-like protein containing a CARD (ASC) and caspase 1, builds the inflammasome that leads to the proteolytic maturation of IL-1β and IL-18 cytokines and inflammation [43,44]. In addition, the activated caspase 1 cleaves gasdermin D (GSDMD) leading to pyroptosis, a highly inflammatory form of apoptosis [45]. Interestingly, Bauernfried et al. found that human NLRP1, but not murine NLRP1B, could be immunoprecipitated by dsRNA [44].

In eukaryotes, 5’ends of mRNAs consist of m7GpppNm (cap1) or m7GpppN1mN2m (cap2) where Nm is 2’-O-methylated nucleotide. The lack of methylation on cap0 (m7GpppN) can also lead to RIG-I activation, while using cap1 or cap2 decreases the induction of cytokines through the RNA sensors RIG-I and MDA5, improving safety [41,46]. Also, while the interferon (IFN)-induced tetratricopeptide repeat (IFIT) protein 1 (IFIT1), a known translation inhibitor, competes with eIF4E for binding to cap0, it shows a significantly lower affinity to cap1 and cap2 [47,48].

The mechanisms described above suggest that the crude, non-purified IVT reaction containing non-optimized mRNA formulated to LNP-mRNA therapeutics can cause a boost and potential overreaction of the immune response, which may result in a decrease in translation, RNA degradation, and even apoptosis of the targeted cells. Thus, mRNA optimization and purification are crucial steps towards the development of LNP-mRNA with enhanced pharmacological and beneficial safety profile for mRNA-based non-immunotherapy applications.

## 3. LNP Induced Immune Activation

**Figure 2:**
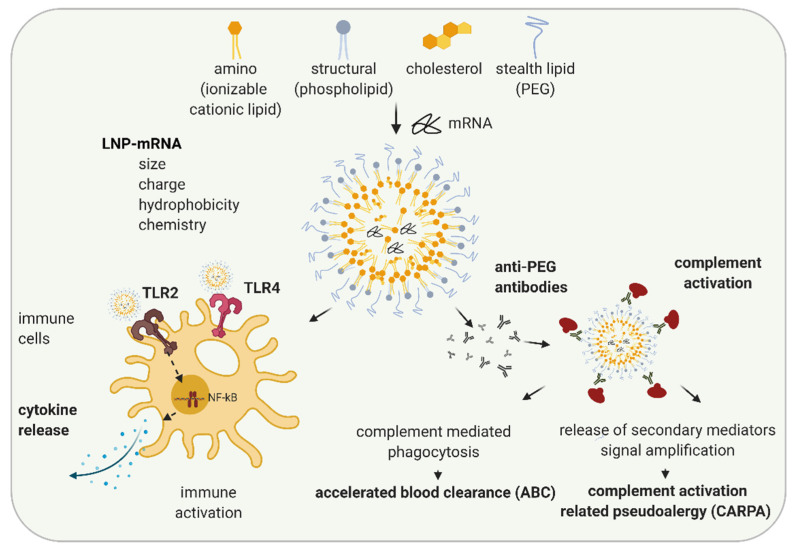
Schematic illustration of LNP-induced immune activation. The lipid nanoparticle (LNP) components, ionizable cationic lipids, structural, stealth lipids, and cholesterol, determine LNP particle size, charge, hydrophobicity, and chemistries. Depending on their features, LNPs can lead to immune activation by activating TLR2 and TLR4 and executing signal transduction pathways leading to NF-kB activation and cytokine secretion. When PEG-lipid is used as a stealth lipid, anti-PEG antibodies can be formed leading to complement activation and subsequently complement mediated phagocytosis resulting in accelerated blood clearance (ABC) phenomenon, or, in rare cases, the release of multiple secondary mediators may occur, leading to complement related pseudoallergy (CARPA). Created with BioRender.com.

Another reason for immune activation by LNP-mRNA is mRNA formulation specificity. While liposomes and lipoplexes were the first formulations applied to mRNA, recently, lipid nanoparticles (LNPs) formulation is widely utilized [49,50]. LNPs were initially developed for the formulation of siRNAs [51,52]. In 2018, the first LNP containing drug, Onpattro^®^, the LNP-siRNA orphan medicinal product for the treatment of transthyretin-mediated amyloidosis, was approved [53,54]. LNPs typically consist of four components: ionizable cationic lipids, structural lipids, cholesterol, and stealth coating lipids (Figure 2). Amino or ionizable cationic lipids are pH-titratable lipids that allow for the entrapment of negatively charged mRNA due to their positive charge under acidic conditions during the formulation process. LNP-mRNAs containing ionizable cationic lipids are non-charged, but are protonated in endosomes with low pH, and help the endosomal release of mRNA by interacting with the negatively charged endosomal lipid bilayer (reviewed in [55,56]). While structural lipids allow the maintenance of the particle structure, cholesterol enhances particle stability, thereby most likely affecting LNP morphology and mRNA delivery [57,58]. Stealth coating lipids such as polyethylene glycol (PEG)-lipid or polysarcosine (pSar) enable the control of physicochemical characteristics of the LNP-mRNA (e.g., particle size and the structure) and influence the circulation half-life of the particle [56,59,60].

Numerous studies have examined structure-activity relationships and revealed that properties of LNP such as particle size, charge, hydrophobicity, components molar fraction, and chemistry of the surface influence LNP interaction with the immune system [61,62,63]. Depending on their characteristics, LNPs can cause various in vivo immune effects: activation of immune cells, inflammation, adaptive immune response, and in some cases, complement activation and complement activation-related pseudoallergy (CARPA) (Figure 2) [64,65,66]. Cationic lipid nanocarriers are recognized by TLR2 and TLR4 located on the cell surfaces of macrophages and other cells [67,68,69]. LNP-TLR’s recognition triggers cytokine and chemokine secretion through similar pathways, as previously discussed for RNA-TLR recognition. Abrams et al. showed the induction of interleukin 1α (IL-1 α), IL-1 β, IL-6, IL-10, and TNF-α after the intravenous (i.v.) application of 0.5–8 mg/kg LNP with or without siRNA to mice, indicating LNP components as primarily responsible for the observed innate immune response [70]. In the same study, more than 10-fold upregulation of at least one-third of 91 tested pro-inflammatory genes were observed indicating inflammation. Cationic lipid nanocarriers can also activate the NLRP3 component of the inflammasome and lead to inflammation [67].

In most cases, currently used LNPs contain PEG lipids. PEG lipids sterically shield LNPs from interacting with other lipid particles or blood components, thus lowering LNP self-aggregation, opsonization, or phagocytosis [71]. PEG is widely used in cosmetics and the food industry. Immunogenicity of PEG is known since 1983, when the injection of PEGylated protein caused the production of anti-PEG antibodies in rabbits [72]. The wide usage of PEG in different industries leads to an increase in the percentage of healthy volunteers positive on anti-PEG antibodies, from 0.2% in 1984 to about 40% in 2016 (reviewed in [65,73]). Recently, a number of studies have shown the formation of anti-PEG IgM and, to a lower extent, IgG antibodies against LNPs and liposomes containing PEG-lipid in animal studies and in patients [65]. Anti-PEG antibodies hamper the efficacy of LNP-mRNAs, especially upon repetitive dosing (as required in non-immunotherapy applications) and can lead to increased safety risks [73]. The accelerated blood clearance (ABC) phenomenon was established as a term after Dams et al. revealed that the second dose of PEG-liposomes was rapidly cleared from the bloodstream of rats and rhesus monkeys while the first dose exhibited long circulation in the blood [74]. The ABC phenomenon depends on the time interval between applications (reviewed in [75]). For example, while in most of the studies, a 7-days application interval of PEGylated nanoparticles leads to the strong ABC after the second dose, a 28-days interval leads to a significantly less clearance [75]. Except the time interval between injections, numerous other factors affecting ABC phenomenon, e.g., animal species, chemical and physicochemical properties of LNP, and dependance on encapsulated drug are established (reviewed in [65,75,76]).

In a recent study on pigs, Kozma et al. examined how PEG-coated liposomes affect hypersensitivity reactions (HSRs) and found that the binding of anti-PEG IgM antibodies to PEGylated liposomes can lead to complement activation and CARPA [77]. CARPA represents the major mechanism of infusion reactions of which the pseudo-anaphylactic shock is the worst outcome. However, the development of infusion reactions highly depends on PEG characteristics as well as on immune system variability and previous PEG exposure in individuals. By now, more than 15 drugs with prominent examples, such as Doxil^®^, Onpattro^®^, BioNTech/Pfizer COVID-19 vaccine (COMIRNATY^®^), and MODERNA COVID-19 VACCINE^®^, containing PEG, are approved or have emergency authorizations by regulatory agencies [64]. These drugs are in use since the benefits of their application significantly outweigh the potential safety concerns, the majority of which originate from infusion reactions. While, typically, infusion reactions cause minor side effects (e.g., headache and muscle pain) that do not require further medical treatment, in rare cases, they can cause anaphylaxis and require treatment with epinephrine. Further studies and a better understanding of the mechanisms of immune activation and sporadic hypersensitive reactions that may be caused by LNP-mRNAs are needed.

## 4. LNP-mRNA in RNA Protein Replacement and Other Non-Immunotherapy Applications

While an increase in the efficacy of the drug, allowing an improved clinical outcome as well as the lowering of dose and costs is a common aim of preclinical studies, safety is a prerequisite. Maximal safety through a decrease in potential side effects of LNP-mRNA applications is essential for RNA protein replacement therapies, including rare disease and other non-immunotherapy approaches. These LNP-mRNA-based applications typically require repetitive dosing through prolonged time-periods (e.g., until organ transplantation) or over a lifetime. Moreover, the introduction of relatively large doses, and, in some cases (such as those for rare inherited disease applications), targeting the already diseased organs, often early in life is needed. Current preclinical and clinical RNA-based protein replacement therapies (RPRTs) are summarized herein. The safety considerations described here for RPRT applications also apply for all LNP-mRNA-based non-vaccine and non-immunotherapy applications such as monoclonal or bispecific antibody therapies used in oncology or infectious disease settings [78,79].

Compared with vaccines and immunotherapy applications, LNP-mRNA non-immunotherapy applications require a larger set of preclinical tests with a strong focus on safety. Based on the previously discussed mechanisms of immune activation by LNP-mRNAs, preclinical tests of such studies would optimally include: (1) correlation of increasing LNP-mRNA dose with cytokine and chemokine secretion, (2) complement activation, (3) repeated administration effects with examination of anti-drug antibodies, (4) acute liver toxicity markers and potential lipid accumulation, and (5) histopathology of the targeted organ.

Unfortunately, the current literature on LNP-mRNA non-immunotherapy preclinical studies is mainly focused on drug efficacy in mouse models and offers limited safety data (Table 1). The listed preclinical studies covering recently published studies on RNA protein replacement show that this is still a relatively young field. Kormann et al. were the first to apply naked modified mRNAs encoding surfactant protein B (SP-B) and erythropoietin in the context of RNA protein replacement therapy in 2011 [80]. However, the first study using LNP-formulated mRNA for RPRT was published only in 2016 (Table 1). In that study, Nabhan et al. applied LNP-mRNA encoding human frataxin as a potential therapeutic against Friedreich’s ataxia [81]. The majority of the studies were published only recently, in the last 3–4 years (Table 1). Although all of these studies show therapeutically relevant amounts of proteins of interest produced after the introduction of corresponding LNP-mRNA, less than a half of the 20 listed preclinical studies address safety issues.

Traditionally, toxicological in vivo studies include examination of liver toxicity by measurement of clinical chemistry markers, e.g., alanine aminotransferase (ALT) and aspartate aminotransferase (AST), plus histopathology of immune and/or target organs. Depending on the route of drug application and specificities of the LNP, diverse organs can be targeted by LNP-mRNA therapeutics, with the liver as the most often targeted organ. Consistent with most of the studies listed in Table 1, safety was traditionally examined through liver toxicity and histopathology studies. However, while traditional safety studies can detect strong impairments of the immune system, they would not deliver information regarding moderate and low immunotoxicity. Thus, additional studies addressing cytokine and chemokine secretion, ADAs, as well as complement activation are required to predict potential safety concerns such as immune activation or impairments.

Cytokines and chemokines are biomarkers of immunotoxicity [101]. They have numerous functions in regulating immune responses and are a known cause of infusion reactions that can be characterized by fever, hypotension, vomiting, chills, headache, nausea, muscle pain, and so on [102,103]. Cytokines, namely, interferon (IFN) gamma, interleukin (IL)-1, IL-2, IL-6, IL-1β, and tumor necrosis factor (TNF), typically lead to pro-inflammatory effects. IL-1 and IL-6 cause fever, and TNFα hypotension and IFNγ activate macrophages further boosting cytokine/chemokine secretion. Chemokines such as interferon-gamma induced protein 10 kD, CXCL10 (IP-10), macrophage inflammatory protein-1 alpha (MIP-1α), or MIP-1 Beta (MIP-1β), and monocyte chemoattractant protein-1 (MCP-1) play a role in leukocyte recruitment and trafficking and may have a role in hyperinflammation when dysregulated, as reviewed in [103].

Interestingly, physiological levels of cytokine and chemokine in the serum of healthy volunteers depend on age and show individual differences [104]. While some cytokines/chemokines such as IL-2 show relatively constant levels through the population, others such as IP-10 and MCP-1 can show 3–4-fold differences between individuals [104]. These basal physiological differences in the immune systems of healthy subjects may contribute to the diversity of responses to drug candidates. While the majority of patients would not develop infusion reactions, some may have mild to moderate adverse effects. In rare cases, life-threating conditions caused by large cytokine release called cytokine storm may occur [103].

For LNP-mRNA drug candidates, U.S. Food and Drug Administration (FDA) recommends preclinical in vitro studies in human whole blood or peripheral blood mononuclear cells (PBMCs) and in vivo animal studies testing broad cytokine/chemokine panels including IL-2, IL-6, IFNγ, and TNFα, in order to map out the potential of exacerbated infusion related toxicities [105]. Interestingly, FDA also recommends that signs of cellular activation in vitro in human cells should be taken as a predictor of potential toxicities in the clinic regardless of negative findings from animal studies [105]. The reason for such a statement originates from 2006, when the therapy with monoclonal antibody TGN1412 passed safety preclinical tests including cynomolgous study, but ended up in a cytokine storm about 1 h after the drug application in all six subjects, resulting in multiple organ failures in two human subjects in the Phase 1 clinical study [106]. Later, it was found that lack of a specific human T cell receptor in all preclinical animal models led to such a misjudgment of drug safety. In vitro studies using human PBMCs added to the immobilized mAb or co-cultured with endothelial cells and then treated with mAb could help to predict the outcome through the detection of TNFα, IL-2, IL-6, IL-8, and IFNγ cytokine release [106].

Table 2 provides an overview of the few studies that have examined cytokine/chemokine secretion in current RNA protein replacement preclinical studies literature. There are various aspects to be considered when evaluating preclinical cytokine and chemokine secretion data: (1) the animal model used and availability of in vitro human data; (2) applied dose and, for in vivo studies, the route of administration; (3) evaluation after single or repetitive dosing, including time of the evaluation; (4) cytokine/chemokine panel tested; and (5) assay used. In all listed studies, only in vivo mouse samples were tested, whereas human in vitro data were lacking. Additionally, only the results after at least the third dose of repetitive dosing were obtained, while the effects of the single dose were not examined. In the study of An et al., only data after the application of a low dose (0.2 mg/kg) and at a late (24 h) time was available, which showed no cytokine secretion [85]. Ramaswamy et al. and Prieve et al. tested cytokines at 3–7 h upon the application of a relatively high LNP-mRNA dose and found significant secretion of Granulocyte-Colony Stimulating Factor (G-CSF), MCP-1, MIP-1β, IL-6, RANTES, and IL-12 (Table 2) [83,86].

In addition to the disease-related studies summarized here, only a limited number of other LNP-mRNA studies examined safety in more detail. Sedic et al. examined the safety of LNP-mRNA encoding for erythropoietin (EPO) in rats and non-human primates (NHPs) [107]. In rats, upon i.v. application of 0.3 mg/kg once or twice weekly, IP-10 was elevated 6 h and 24 h post-dose, while no change in IL-6, TNFα, and IFNα was observed. In monkeys, no change in tested cytokines/chemokines was detected when the same dose was applied. In the same study, elevation of C3a and C5b-9 with the magnitude increasing with repeated dosing was found in monkeys, whereas no complement activation could be observed in rats [107]. While testing novel amino lipid components of LNPs, Sabnis et al. also performed toxicology evaluation including liver toxicology, complement and MCP-1 serum concentration in cynomolgous monkeys infused with 1 mg/kg LNP-mRNA at 2 h, 6 h, and 25 h after day 1 and day 29 [108]. The authors found no indication for liver toxicity or complement activation, while a slight increase in MCP-1 at 2 h after the application on day 1 was observed. This effect became negligible at day 29. Unfortunately, the safety studies were shown only for the most effective novel amino lipid LNP5 while it stayed unclear how they compare for other tested lipids including the control MC3 lipid that was previously used in LNP-siRNA approved drug, Onpattro^®^ [53]. Magueri et al. analyzed mouse plasma cytokines: regulated upon activation, normal T cell expressed, and secreted (RANTES), keratinocytes-derived chemokine (KC), IL-6, IP-10, IL-1β, TNFα, MCP-1, and IFNγ, 5 h and 24 h after i.v. injection of 1.5 µg of LNP containing erythropoietin mRNA [109]. Interestingly, in this study, the authors found that cytokine concentrations were higher when LNP-mRNA was injected, compared with the injection of the same mRNA packed in extracellular vesicles (endo-EV-mRNAs) naturally formed upon the secretion of that endocytosed LNP-mRNA [109]. Recently, Noguiera et al. compared the safety profiles of LNPs with different stealth coating lipids: polyethylene glycol (PEG)-lipid and polysarcosine (pSar) [59]. The authors tested IL-8, IL-6, IL1-β, IFN-γ, TNF-α, IL-2, IL-10, IL-4, IL-5, and granulocyte-macrophage colony-stimulating factor (GM-CSF) in human plasma from whole blood and found that LNPs formulated with pSar23 showed a reduced cytokine profile, compared with those prepared with 1,2-Dimyristoyl-rac-glycero-3-methylpolyoxyethylene (PEG-DMG). In the same study, liver toxicology markers AST, ALT, laktat-dehydrogenase (LDH), and total Bilirubin were examined in mouse model upon weekly multiple injections during four weeks and 48 h post last injection, where comparable or advantageous safety profile was detected for pSar containing LNP [59]. Moreover, the authors incubated pSar23 and PEG LNPs with a human serum and found lower induction of C3a complement levels when a high dose of pSar23 was used compared with PEG LNP, indicating the lower toxicity of LNP formulated with pSar [59].

While these safety studies provide limited data on immune activation by applied LNP-mRNA drugs, there is a clear need for additional data that would address the following: the comparison of model systems and assays, as well as establishing optimal predictive panels, new biomarkers, and optimal testing time. Moreover, a better understanding of ranges and cut offs in cytokine/chemokine preclinical measurements and mapping out differences in between human donors would all together increase the predictive value of preclinical studies for clinics.

Currently, there are only a few ongoing LNP-mRNA clinical studies for RNA protein replacement (Table 3). From studies that were started between 2018–2020, two were discontinued: MRT5201 due to program discontinuation and mRNA-3704 due to a business decision. Phase 1 clinical trial of ARCT-810, the drug candidate for OTC deficiency, was successfully completed in healthy adults and is currently recruiting in a Phase 1/2 OTCD study. Similarly, the NCT03375047 study that examines the MRT5005 Cystic Fibrosis drug candidate is currently recruiting in Phase 1/2.

Moreover, in addition to the clinical studies discussed here, companies such as Arcturus, Translate Bio, CureVac, BioNTech/Genevant, and Moderna have multiple LNP-mRNA-based RPRT/rare disease drug candidates in their pipelines (Table 4). With more LNP-mRNA drug candidates entering the clinic, examining the correlation between preclinical and clinical data would become possible. This would allow a better defining of preclinical regulatory guidelines. Finally, this would also allow a better understanding and use of the predictive value of preclinical models for further improving LNP-mRNA drug efficacy and safety, especially for non-immunogenic LNP-mRNA applications.

## 5. Boosting the Efficacy and Safety of LNP-mRNA Applications

**Figure 3:**
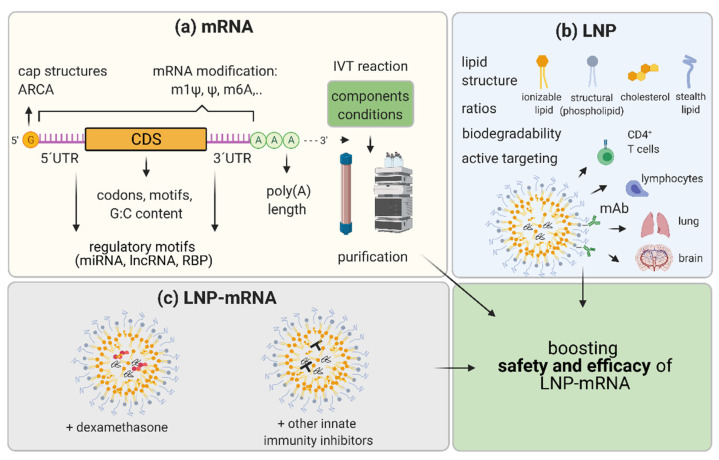
Representation of the strategies to boost efficacy and safety of LNP-mRNA. (**a**) mRNA optimization consists of (1) using different mRNA nucleoside modifications; (2) increasing capping of mRNA by varying cap structures and/or utilizing anti-reverse cap analogues (ARCA); (3) selection of most efficacious UTRs: optimization of 5′UTRs and 3′UTRs by avoiding or exploiting regulatory motifs recognized by micro (mi)RNAs, long non-coding (lnc)RNAs, and RNA binding proteins (RBPs); (4) optimization of the coding sequence (CDS) by using more frequent codons, avoiding specific motifs, and varying G:C content; and (5) varying components and conditions of in vitro transcription (IVT) reaction to decrease byproducts and increase yield, and developing optimal purification protocols for diverse applications. (**b**) Lipid nanoparticle (LNP) optimization may include (1) changing chemistry and molar ratios in between LNP components with a focus on biodegradability and novelty in ionizable lipids and stealth lipids; and (2) the active targeting of other tissues such as using monoclonal antibodies (mAb) recognizing specific molecules on T cells, lymphocytes, and brain or lung vasculature. (**c**) Moreover, to achieve increased efficacy and maximal safety, co-formulating molecules as corticosteroids (dexamethasone) or other small molecules known to inhibit innate immunity with LNP-mRNA candidate therapeutics may be performed. Optimization of the mRNA and LNP as components and combining LNP-mRNA with immune inhibitors aim to boost safety and efficacy of LNP-mRNA drug candidates. Created with BioRender.com.

Innovation in mRNA and LNP components of LNP-mRNA drug candidates and improvements in the methods of their production are hallmarks of this relatively young therapeutic field. This constant development is the basis for the enormous therapeutic potential and expected growth in LNP-mRNA applicability not only for vaccines and immunotherapy, but also for more challenging applications such as RNA protein replacement and monoclonal antibody therapies. Innovation at the mRNA level includes (1) RNA nucleoside modification, (2) sequence and structure optimization, and (3) IVT mRNA production and purification methods (Figure 3).

### 5.1. mRNA Nucleoside Modification

mRNA nucleoside modifications were a key finding that led to a boost in the efficacy and safety of mRNA. Karikó et al. discovered, in 2005, that nucleoside-modified RNA is far less immunogenic, compared with unmodified mRNA [13]. The study showed that the incorporation of modified nucleosides 5-methylcytosine (m5C), 6-methyladenosine (m6A), 5-methyluridine (m5U), 2-thiouridine (s2U), or pseudouridine (Ψ), when compared with unmodified RNA, significantly reduced the secretion of cytokines by human dendritic cells (DCs). Increasing the content of the modified nucleosides per mRNA was directly proportional to the relative inhibition of TNFα expression in DCs. In 2008, Karikó et al. performed mouse in vivo studies and found that not only the safety, but also the translational capacity and mRNA stability were increased when RNA was modified [110]. Andries et al. tested translation and immunogenicity in vitro and in vivo in mice and found that 1-methylpseudouridine (m1Ψ)–incorporated mRNA outperforms Ψ- incorporated ones [111]. Due to its ability to significantly decrease probability for infusion reactions and to increase the efficacy, today, m1Ψ has become the most commonly used RNA modification for various LNP-mRNA applications: vaccines, therapeutic antibodies, or RNA protein replacement [9,78,83,112,113]. For example, BioNTech/Pfizer vaccine as well as the Moderna vaccine against COVID-19 utilize m1Ψ- modified mRNA [9,114].

### 5.2. mRNA Sequence and Structure Optimization

The mRNA sequence and structure optimization include a panel of strategies known to improve pharmacology and the safety of LNP-mRNA therapeutics. Optimizing cap structure, 5′ and 3′UTRs, coding sequence, and poly(A) tail length may significantly influence the performance of LNP-mRNA therapeutics (Figure 3). Efficiently linking 7-methylguanosine (m7G) cap to the synthetic mRNA by 5’-5’triphosphate bridge and forming m7GpppN structure is necessary for efficient translation [115]. In the cytoplasm, the eIF4E translation initiation factor binds to the cap allowing the start of mRNA translation [116,117]. Together with the poly(A) tail and RNA binding proteins, the cap is crucial for mRNA circularization, which ensures full-length translation and translation enhancement [118]. Additionally, in the cytoplasm, the cap binds mRNA decapping machinery, thus influencing mRNA degradation [119]. It was previously discussed that cap0, but not cap1, induces cytokines through RIG-I and MDA5 and that IFIT1 can bind to cap0 but with significantly lower affinity to cap1 or cap2 [41,46,47,48]. Thus, it was expected that the presence of methylation on cap1 can also improve translation efficacy in certain cell types [120]. In the last 20 years, diverse synthetic cap structures were developed to enhance the efficiency and safety of IVT mRNA. Cap can be enzymatically added to the mRNA 5′ end using vaccinia capping enzyme to form a cap0 following Vaccinia 2′ O-methyltransferase application to finalize the cap1, as recently used in Modernas’ COVID-19 vaccine mRNA-1273 [114]. Alternatively, the cap can be added during IVT reaction in a process called co-transcriptional capping, as in BioNTech/Pfizer COVID-19 vaccine BNT162b2 [114,121,122,123,124]. Recently, diverse types of trinucleotide cap1 analogues allowing co-transcriptional capping are commercially available. CleanCap Cap1 AG trimer, and anti-reverse cap analogue (ARCA) CleanCap1 are widely used [120,124,125].

Another important structural feature of mRNAs, which can define their stability, localization, and expression are untranslated regions (UTRs) located on the 5′ and 3′ end of mRNAs (5′UTRs and 3′UTRs) [126,127]. They exhibit cis-regulatory elements in their sequence recognized by microRNAs (miRNAs), long ncRNAs (lncRNAs), or RNA Binding Proteins (RBPs) that impact translation and determine the fate of mRNA. Jain et al. introduced miRNA target sites to UTRs of therapeutic mRNAs to recruit endogenous miRNAs, thereby reducing the off-target expression of mRNA [128]. In their study, the introduction of multiple copies of hepatocyte specific miR-122 target sites to 3′UTRs of the mRNAs encoding apoptotic proteins prevented mRNA expression in healthy hepatocytes while allowing selective apoptosis in hepatocellular carcinoma cells. Exploiting post-transcriptional regulation of mRNA therapeutics by cellular lncRNAs and RBPs is still in its infancy and certainly will be an interesting field of innovation in the future. 5′UTR structures such as hairpins, pseudoknots, RNA G-quadruplexes, upstream open reading frames (uORF), and upstream start codons (uAUGs) that overall inhibit translation should be avoided when engineering optimal 5′UTRs for prolonged expression of LNP-mRNA therapeutics [127].

The most widely used 5′ and 3′UTRs for therapeutic mRNAs are those from α- and β-globin mRNAs that contain elements, which increase mRNA translation and stability [129,130]. Multiple studies screened optimal UTRs for diverse applications. For example, Asrani et al. indicated 5′UTR as a key driver in protein expression and, in a screen of ten 5′UTRs, revealed that the complement factor 3 (C3) and cytochrome p4502E1 (CYP2E1) 5′UTRs demonstrated the largest and most consistent increase in protein expression relative to a reference UTR in vitro in human cells [131]. Sample et al. recently built a library of 280,000 randomized 50mer 5′UTRs that they combined with polysome profiling and deep learning. Subsequently, they used this to build a model and to engineer new 5′UTRs that can direct ribosome loading and provide optimal translation [132]. Motifs of 3′UTR were recently screened by Orlandini von Niessen et al., exploiting an unbiased in vitro method where motifs were correlated with mRNA stabilizing activity and activity in promoting high translation. This screen found that using the amino-terminal enhancer of split (AES)-mitochondrially encoded 12S rRNA (mtRNR1)-based 3′UTR elements were beneficial over the previously used two head-to-tail-cloned human β-globin 3′UTRs (2hBg) in different systems including mice after i.v. vaccination with gp70-encoding mRNAs [133]. Those 3′UTR elements were recently exploited in the BioNTech/Pfizer vaccine against COVID-19 [125].

Engineering the optimal coding sequence by replacing rare codons with frequently used synonymous codons, increasing G:C content, and avoiding certain regulatory sequences overall increase the mRNA protein expression (reviewed elsewhere [134,135,136]). However, diverse codon optimizations must be tested empirically depending on the therapeutic mRNA application and specific targeted cell type. The Poly(A) tail, together with a cap, has an impact on translation and mRNA stability [137]. The poly(A) tail can be defined in a DNA plasmid template and transcribed during IVT reaction assuring uniform poly(A) tail length, or, mRNA can be extended after IVT by using recombinant poly(A)polymerase [138]. Both approaches of tailing have limitations: technical difficulties during cloning of plasmids coding long poly(A) tails or, in the case of enzymatic polyadenylation, assuring consistent poly(A) tail length and product uniformity during manufacturing. Today, most therapeutic mRNAs have poly(A) tail lengths of at least 50nt to ≥ 100 nt. These lengths resemble the average lengths of most endogenous mRNAs according to various genome-wide poly(A) tail profiling methods, which revealed that the majority of mRNA tails are significantly shorter than the previously thought tail length of 250 adenosines [139,140,141].

### 5.3. IVT mRNA Production and Purification Methods

The whole manufacturing process of IVT mRNA is a field of constant innovation and optimization with the aim to minimize the level of dsRNA and other contaminants and thus allowing the low immunogenicity of mRNA therapeutics. For example, Wu et al. used high temperature and thermostable T7 RNA polymerase to produce mRNA showing reduced immunogenicity without the need for purification [142]. However, various purification methods are widely utilized in order to reach maximal purity of single-stranded mRNA. For example, ion pair reversed-phase high-performance liquid chromatography (HPLC) is still considered as a gold standard method for the depletion of unwanted byproducts/contaminants from mRNA of interest [143]. Unfortunately, HPLC is difficult to scale up for the manufacture of large amounts of mRNA and would lead to the production of high amounts of hazardous waste [17]. Therefore, cellulose chromatography was recently developed [17]. This method showed a great efficiency in the depletion of dsRNA contaminants and the production of mRNAs with high purity and low immune activation potential.

### 5.4. LNP Optimization

In addition to optimizing mRNA and its manufacturing, optimization of LNP also has a great potential to significantly improve the safety and efficacy of LNP-mRNA-based therapeutics. Specific topics that may be differentiated in this field are (1) innovation in terms of ionizable lipids and biodegradable lipids for different application routes; (2) LNP-mRNA composition optimization; (3) innovations in stealth lipids; and (4) achievement of a specific cell/organ targeting through LNP-based changes (Figure 3b). In this review, these topics are shortly summarized while they are reviewed in more depth elsewhere [4,56].

Ionizable amino lipids are the major LNP component influencing the efficacy and tolerability of LNP-mRNA drugs. They function in cellular uptake, endosomal escape, and LNP ability to non-specifically bind serum proteins to the LNP surface. The first clinically approved amino lipid was MC3 (DLin-MC3-DMA) [53]. However, this lipid is known to have a long half-life in the organism, leading to mild-to-moderate adverse effects in clinical studies, thus being suboptimal for repetitive dosing applications [144,145]. Therefore, novel ionizable and fully biodegradable lipids are constantly being developed. For example, Maier et al. used MC3 as a basis for developing a set of new biodegradable lipids of which L319 showed rapid clearance from plasma and tissues. In addition, L319 was well tolerated based on serum chemistry and histopathology when administered up to 10 mg/kg dose as a single bolus injection in a preclinical setting [144]. Sabnis et al. used a rational medicinal chemistry approach to optimize amino lipids and found a structure named LNP5 showing favorable pharmacokinetics, expression profile, endosomal escape efficiency, tissue clearance, and tolerability in mice and cynomolgous [108]. While both described studies focused on intravenous administration (i.v.), Hasset et al. focused on the optimization of LNP for intramuscular application (i.m.) and screened 30 novel ionizable biodegradable lipids [62]. The authors detected application route-dependent differences during the primary screen of immunogenicity and expression. Five novel propriety lipids that lead to the highest expression of LNP-mRNA in combination with low immunogenicity were applied i.m. in mice, rats, and NHP [62].

Optimization of LNP-mRNA composition includes varying lipid ratios or lipid-to-mRNA ratios. To optimize LNPs for mRNA delivery to liver, Kaufmann et al. developed a Design of Experiment (DOE) methodology [146]. By increasing ionizable lipid:mRNA weight ratios and incorporating 1,2-dioleoyl-sn-glycero-3-phosphoethanolamine (DOPE) as a helper lipid, the authors significantly increased the efficiency of erythropoietin mRNA loaded LNP compared with the control LNP-mRNA based on LNP formulation used for LNP-siRNA delivery [146]. Sago et al. formulated multiple LNP libraries (in total >250 LNPs) varying the amino lipid compound, molar amount, and the structure of PEG, as well as the molar amount of cholesterol [147]. Authors co-formulated Cre mRNA and DNA barcodes to each LNP and injected them i.v. or i.m. to Lox-Stop-Lox-tdTomato (Ai14) mice. They tested the delivery of LNP libraries in vivo based on the translation of Cre mRNA to Cre protein and isolation of fluorescent cells where targeted DNA was edited by Cre protein. This approach resulted in the identification of two novel LNPs that efficiently deliver mRNA to endothelial cells [147]. This study showed the importance of optimization of LNP composition not only for improvements in efficacy but also as a path for identifying LNPs with new tropisms.

Stealth lipids such as PEG-lipid are necessary for increased half-life and stability of the LNP particle and influence its physicochemical properties. After LNP-mRNA administration to the blood, LNP adsorbs on its surface numerous proteins forming “protein corona” (reviewed in [63,148]). Among others, these proteins include albumin, immunoglobulins, lipoproteins, apolipoproteins, coagulation factors, and complement proteins [149]. PEG-lipids-shielding lowers the interaction of LNP with complement and other proteins lowering the internalization of LNP-mRNAs by macrophages increasing circulation time of LNP-mRNA in the blood. In addition, PEG-lipids-shielding has impact against undesired aggregation and accumulation in filtering organs that might be caused by protein corona [148]. However, PEG-shielding may also lower the recognition of apolipoprotein E (ApoE), and it can cause the formation of anti-PEG ADAs that lead to lowering the efficiency of the LNP [60,150]. Thus, the level of PEG-shielding must be optimized to obtain a compromise between efficacy and safety. With regard to anti-PEG ADAs formation, a recent study by Suzuki et al. examined PEG-containing LNP-siRNA in mice and found that LNPs with a fast-shedding PEG-lipid (short acyl chain) induced less anti-PEG IgM compared with those with long acyl chain LNPs [60]. The usage of the fast-shedding PEG-lipid allowed more hepatocyte targeting compared with Kupffer cells, the liver macrophages, thus improving the effectivity of LNP-siRNA drug [60]. This study is in agreement with the previous study by Judge et al. where the authors found less formed anti-PEG antibodies and a substantial reduction of side effects upon repetitive dosing in mice when PEGylated liposomes containing a shorter alkyl chain (C14) PEG-lipid versus a longer alkyl chain C16 PEG-lipid were used [151]. Studies directly examining the effects of anti-PEG antibodies on the efficacy and safety of LNP-mRNA drugs containing PEG lipids are still very limited. Recently, Nogueira et al. examined diverse chain lengths and molar fractions of stealth lipid Polysarcosine (PSar) and found efficient mRNA delivery with a lower cytokine pro-inflammatory profile, reduced complement activation, and liver toxicity markers, compared with PEG-containing LNPs [59].

Localization to particular tissues and the active targeting of LNP-mRNA therapeutics to specific cell types and organs are a topic of particular interest that can improve current off-target effects and pave a route for novel applications in difficult-to-target tissues. As already discussed, localization to particular tissues can be achieved by optimization on the mRNA level by introducing cell type specific miRNA target sites to 3′UTRs leading to the degradation of the mRNA, leading to the loss of translation efficacy of LNP-mRNA in selected cell types [128]. However, optimization on the level of LNP is the main focus with diverse approaches based on changing LNP structural components and optimizing LNP composition or those actively targeting specific cells using a functionalized surface, for example, with targeting ligands or antibodies. Most of the currently developed LNPs largely localize to the liver through apolipoprotein E (ApoE)-mediated uptake [152]. ApoE binds to LNP in circulation and facilitates binding to low density lipoprotein receptor (LDLR) on hepatocytes, allowing the endocytosis of LNP-mRNA to the cell. Thus, most of the currently existing preclinical and clinical RNA protein replacement, as well as rare disease studies, consider liver diseases or utilize the liver as a protein production factory by using classical LNP formulations of smaller diameter (<100 nm) that are naturally accumulating in hepatocytes. That the size of the LNP particle > 100 nm may be a limiting factor for hepatocyte targeting, especially in humans, could be inferred from Wisse at al., who measured the sizes of fenestrae [153]. Fenestrae are the pores in liver sinusoids with the size of 107 ± 1.5 nm in humans without liver pathology and with a significantly larger size in rodents: C57BL/6 mice (141 ± 5.4 nm) and Sprague–Dawley rats (161 ± 2.7 nm) [153]. To reach hepatocytes, LNP-mRNA must pass through the fenestrae, thus limiting the size of the LNP-mRNA to about 100 nm for hepatocyte targeting.

The localization of LNP to other organs typically requires the optimization of LNP or active targeting. The importance of LNP composition optimization in screening carried out by Sago et al. resulted in finding 7C2 and 7C3 LNPs that efficiently target endothelial cells, as previously noted [147]. Using the same screening methodology as Sago et al., Gan et al. recently tested a library of 109 LNPs composed of “constrained phospholipids” that contained an adamantylhydrocarbon chain [154]. This study identified a novel LNP that delivers mRNA to Kupffer cells instead of hepatocytes, without targeting ligands [154]. Recently, Zukancic et al. used Onpattro^®^ LNP, where the authors replaced 1, 2-Distearoyl-sn-glycero-3-phosphoethanolamine-Poly(ethylene glycol) (PEG-DSPE) with Tween 20 containing short (C11) PEG alkyl chain [155]. The authors found that the usage of the short PEG alkyl chain led to a significantly improved lymph node targeting after intramuscular administration in mice [155]. Few studies focused on actively targeting of lymphocytes. Ramishetti et al. functionalized the LNP surface by anti-CD4 monoclonal antibody to target CD4+ T cells [156]. Veiga et al. have used an ASSET (Anchored Secondary scFv Enabling Targeting), in which anti-Ly6c mAb is linked to LNPs in order to target Ly6c+ inflammatory leukocytes [157]. The authors tested this strategy applying anti-Ly6c mAb coated or isotype control LNP-formulated IL-10 mRNA in a dextran sodium sulfate (DSS) colitis mice model of inflammatory bowel disease, where they showed the beneficial effects of the LNP-mRNA targeted vs. non-targeted approach. Recently, Ramishetti et al. synthesized a set of novel ionizable lipids, used them for mRNA formulation, screened LNP-mRNA expression and safety in leukocytes, and actively targeted primary lymphocytes using β7 integrin [158]. In order to actively target the inflamed brain tissue, Marcos-Contreras et al. tagged anti-vascular cell adhesion molecule 1 (VCAM) mAb to LNP-formulated thrombomodulin mRNA [159]. VCAM is highly expressed in cerebrovascular endothelium and VCAM-targeted LNP-thrombomodulin mRNAs accumulated in the TNFα injured brain mouse model and reduced brain edema caused by the TNFα injection [159]. Similarly, anti-vascular cell adhesion molecule, PECAM-1 mAb, was used for LNP-mRNA targeting lungs and, there, leading to a ~200-fold enhanced delivery, when compared with untargeted tissues [160].

If upon optimization of both mRNA and LNP, components of LNP-mRNA undesired immunostimulatory features persist, one further possibility for their suppression and lowering of potential adverse effects is the incorporation of potent corticosteroids directly into the LNP-mRNA drug product (Figure 3c). Chen et al. incorporated dexamethasone, a potent corticosteroid into the LNP containing various types of nucleic acids [161]. They used biodegradable linkers to chemically conjugate lipophilic acyl/alkyl moieties to dexamethasone and synthesized dexamethasone prodrugs which could be effectively incorporated into the LNPs. The usage of LNP-mRNA containing 10 mol% dexamethasone strongly ameliorated immune stimulation, leading to a significant decrease in IL-6, TNFα, IL12p70, IL-1β, IL-10 and keratinocyte chemoattractant (KC)/human growth-regulated oncogene (GRO) (KC/GRO) in plasma, 4 h after i.v. injection at a mRNA dose of 3 mg/kg in mice. Interestingly, the immunosuppressive effect of the incorporated dexamethasone was significantly higher compared with the free dexamethasone that was co-administered with LNP-mRNA therapeutic [161]. Other strategies to prevent potential unwanted immunostimulation by LNP-mRNAs are the use of other small molecules or siRNAs against key innate immunity response mediators (as reviewed recently by [2,162]). However, the effectivity of such innate immune inhibitors was established only in rare specific cases, indicating the potential challenges for wider applicability and the necessity of further studies.

## 6. Conclusions

In the past years, we have witnessed an accelerated growth of RNA-based technologies and their applications. The first LNP-siRNA drug, Onpattro^®^, for the treatment of transthyretin-mediated amyloidosis, was approved in 2018. Recently, two LNP-mRNA vaccines against SARS-CoV-2 virus obtained emergency or conditional marketing authorizations from multiple regulatory agencies worldwide. However, while clinical studies of other prophylactic vaccines are still scarce (e.g., Cytomegalovirus (CMV), Zika, and Rabies), the numerous cancer vaccines and immuno-oncology actively recruiting clinical studies are in Phase 1 or Phase 2. Key leaders in the field of LNP-mRNA technology development such as BioNTech, Moderna, and CureVac have focused on cancer immunotherapy applications in the following indications: melanoma, non-small cell lung cancer (NSCLC), head and neck cancer, triple negative breast cancer, prostate cancer, pancreatic and ovarian cancer, and multiple solid tumors. There are significant similarities in the different mRNA therapeutic fields in terms of LNP and mRNA development and manufacturing. The similarities encompass all the basic commonalities in the sequence and structure of mRNA that should resemble endogenous mRNA and features of LNP that should allow the most efficient transfer to the cells of interest. However, there are also some differences between immunotherapy (vaccines and cancer immunotherapies) and non-immunotherapy (RNA protein replacement and some of monoclonal antibodies therapeutics) applications.

In most immunotherapy applications (infectious disease and cancer vaccines), a boost of the immune system based on natural recognition of synthetic mRNAs and LNP components mimicking viral attack may be beneficial [1,163]. For example, single-stranded antigen coding RNA oligonucleotides were found to induce T helper cells 1 (Th1)-type cytokines and to simultaneously activate an innate immune response in addition to an adaptive immune antigen specific response [15]. Conversely, in non-immunotherapy applications, fine-tuning of the LNP-mRNA components to fully diminish immune activation and increase safety is crucial. Thus, understanding the basic pharmacodynamics and pharmacokinetics of LNP-mRNA non-immunotherapy drug candidates and their interaction with the host immune system is necessary. Therefore, numerous preclinical studies underwent prolonged optimization to ensure a strong focus on safety before entering the clinic. Over multiple years, both immunotherapy and non-immunotherapy mRNA therapeutic fields underwent a great deal of innovation and significant growth. Self-amplifying mRNA (saRNA) encode replicase and protein of interest and replicate in cells utilizing viral replication strategy (comprehensively reviewed elsewhere [164,165]). saRNA generate dsRNA intermediate during their cellular amplification, are potent activators of the immune system, and are one of the innovative tools in immunotherapy. The mRNA modification and other numerous improvements in the domain of conventional mRNA and LNP structure, as well as their manufacturing, were and are continuously being carried out. However, since this is a relatively young field, efforts to better clarify the necessity for collecting not only traditional toxicology and histopathology data, but also extended safety data such as on cytokines/chemokines, complement, and anti-drug antibodies are necessary. These markers are found to be more sensitive and can serve as better predictors of potential adverse effects. To better predict clinical outcomes, similar to their use in the field of small molecule drugs, systems biology and modeling should also be increasingly exploited in LNP-mRNAs preclinical studies. Moreover, a commitment to better understand the predictive value of non-clinical study models and the differences between model systems is needed. Furthermore, developing primary human cells, tissues, and organ cultures as models for measurements of LNP-mRNA therapeutics efficiency and safety would be beneficial.

As summarized, only a few Phase 1 clinical studies are currently ongoing in the RNA protein replacement/rare disease field. Further efforts in optimizing LNP-mRNAs, examining the potential for combination with small molecule drugs or other medical treatments, and improving preclinical and regulatory guidelines will certainly lead to more high-quality preclinical and clinical LNP-mRNA non-immunotherapy studies. Particularly, a large impact is to be expected for the delivery of neutralizing monoclonal antibodies (such as the currently ongoing mRNA-1944 clinical study against Chikungunya virus, NCT03829384) and in the rare disease field where high unmet medical needs are present among many indications.

## Figures and Tables

**Table 1:** Overview of current LNP-mRNA-based protein replacement and rare disease preclinical studies.

Delivery	mRNA	Disease	Company	Safety Data	Reference
LNP	Frataxin (FXN)	Friedreich’s ataxia	Pfizer	no	Nabhan et al., Scientific Reports, 2016 [81]
LNP	Factor IX	Hemophilia B	Shire	no	DeRosa F. et al., Gene Therapy, 2016 [82]
LNP	Factor IX	Hemophilia B	Arcturus	cytokines, liver toxicity, liver histopathology	Ramaswamy S. et al., PNAS ^3^, 2017 [83]
LNP	cystic fibrosis trans-membrane conductance regulator (CFTR)	Cystic Fibrosis	Arcturus, Translate Bio	no	Robinson et al., Mol Therapy, 2018 [84]
LNP	methylmalonyl-CoA mutase (MUT)	Methylmalonic Acidemia	Moderna	cytokines, ADA ^1^, liver toxicity	An D. et al., Cell reports, 2018 [85]
LNP/ HMT ^2^	ornithine transcarbamylase (OTC)	OTC Deficiency	PhaseRx	cytokines, liver toxicity, liver histopathology	Prieve M. et al., Molecular Therapy, 2018 [86]
LNP	porphobilinogen deaminase (PBGD)	Acute intermittent porphyria	Moderna	liver toxicity, ADA ^1^	Jiang L. et al., Nature Medicine, 2018 [87]
LNP	disintegrin and metalloprotease with thrombospondin type 1 repeats, member 13 (ADAMTS13)	Thrombotic thrombocytopenic purpura	Alexion	no	Liu-Chen S. et al., Scientific Reports, 2018 [88]
LNP	uridine-diphosphateglucuronosyltransferase (UGT1A1)	Crigler-Najjar Syndrome Type 1	Alexion	no	Apgar J. et al., CPT Pharmacometrics Syst. Pharmacol, 2018 [89]
LNP	serine protease inhibitor, group A, member 1 (SERPINA1)	Alpha-1 Antitrypsin Deficiency	Alexion	no	Connolly B. et al., Journal of Nucleic Acids, 2018 [90]
LNP	glucose-6-phosphatase (G6Pase)	Glycogen storage disease type Ia	Alexion	no	Roseman D. et al., Molecular Therapy, 2018 [91]
LNP	arginase I (ARG1)	Arginase I deficiency	Alexion	no	Asrani et al., RNA Biology, 2018 [92]
LNP	citrin (aspartate/glutamate transporter)	Citrin deficiency	Moderna	no	Cao J. et al., Molecular Therapy, 2019 [93]
LNP	alpha galactosidase A (a-Gal A)	Fabry Disease	Translate Bio, Shire	no	De Rosa et al., Molecular Therapy, 2019 [94]
LNP	oxysterol 7-a-hydroxylase (CYP7B1)	Hereditary Spastic Paraplegia Type 5	CureVac	liver toxicity	Hauser S. et al., Molecular Therapy, 2019 [95]
LNP	alpha galactosidase A (a-Gal A)	Fabry Disease	Moderna	liver toxicity, ADA ^1^	Zhu et al., The American Journal of Human Genetics, 2019 [96]
LNP	arginase 1 (ARG1)	Arginase deficiency	Moderna	liver histopathology	Truong B. et al., PNAS^3^, 2019 [97]
LNP	methylmalonyl-CoA mutase (MUT)	Methylmalonic Acidemia	Moderna	liver toxicity, liver histopathology	An et al., EbioMedicine, 2019 [98]
LNP	galactose-1 phosphate uridylyltransferase (GALT)	Galactosemia	Moderna	no	Balakrishnan B. et al., Molecular Therapy, 2020 [99]
LNP	serine protease inhibitor, group A, member 1 (SERPINA1)	Alpha-1 Antitrypsin Deficiency	Moderna	liver toxicity, liver histopathology	Karadagi A. et al., Scientific Reports, 2020 [100]

^1^ ADA: anti-drug antibody; ^2^ HMT: Hybrid mRNA Technology delivery system; ^3^ PNAS: Proceedings of the National Academy of Sciences of the United States of America.

**Table 2:** Overview of preclinical studies that have examined cytokine/chemokine secretion in current RNA Protein Replacement Therapies.

Drug Candidate	Animal, Dose, Time	Cytokines/Chemokines	Significant Upregulation Compared to Control	Assay	Reference
LNP-Factor IX mRNA	mouse, 4 mg/kg i.v.; 4 h, 7 h, 24 h and 48 h after third dose	IL-1α, IL-1β, IL-2, IL-3, IL-4, IL-5, MIP-1α, IL-10, IL-12 p40, IL-12 p70, IL-13, IL-17α, G-CSF, GM-CSF, IFNγ, KC, MCP-1, MIP-1β, RANTES, TNFα, IL-6, Eotaxin	yes (4 h and 7 h): G-CSF, MCP-1, MIP-1β, IL-6, RANTES; no (24 h and 48 h)	Biorad multiplex	Ramaswamy S. et al., PNAS, 2017 [83]
LNP-MUT ^1^ mRNA	mouse, 0.2 mg/kg i.v.; 24 h after third or fifth weekly dose	IL-6, IFNγ, TNFα, IL-1β	no (24 h)	MSD ^4^ multiplex	An D., et al., Cell reports, 2018 [85]
LNP/HMT ^2^ OTC ^3^ mRNA	mouse, 3 mg/kg i.v.; 3 h and 24 h after ninth repeat dose	IL-6, IL-12, GM-CSF, IFNγ, TNFα, CXCL10, MCP-1	yes (3 h and 24 h): IL-12	Luminex multiplex, ELISA ^5^ (CXCL10)	Prieve M. et al., Molecular Therapy, 2018 [86]

^1^ MUT: methylmalonyl-CoA mutase; ^2^ HMT: Hybrid mRNA Technology delivery system; ^3^ OTC: ornithine transcarbamylase; ^4^ MSD: Meso Scale Discovery; ^5^ ELISA: enzyme-linked immunosorbent assay.

**Table 3:** LNP-mRNA-based RNA protein replacement clinical studies (as of February 2021).

Candidate	Biological Target	Disease	Company	Year Start	Clinical Phase	Number
MRT5005	CFTR ^1^	Cystic Fibrosis	Translate Bio	2017	Phase 1/2	NCT03375047
MRT5201	OTC ^2^	OTC Deficiency	Translate Bio	2018	Phase 1/2	NCT03767270 (program discontinued)
mRNA-3704	MUT ^3^	Methylmalonic Acidemia	ModernaTX, Inc.	2019	Phase 1/2	NCT03810690 EU 2019-001061-32 (Terminated due to business decision)
mRNA-3927	PCCA and PCCB ^4^	Propionic Acidemia	ModernaTX, Inc.	2019 (US), 2020 (EU)	Phase 1/2	NCT04159103 (not yet recruiting) EU 2019-003529-36
ARCT-810	OTC ^2^	OTC Deficiency	Arcturus	2020	Phase 1	NCT04416126 (completed, healthy adult subjects) NCT04442347 (recruiting)

^1^ CFTR: cystic fibrosis trans-membrane conductance regulator; ^2^ OTC: ornithine transcarbamylase; ^3^ MUT: methylmalonyl-CoA mutase; ^4^ PCCA and PCCB: propionyl CoA carboxylase α- and β.

**Table 4:** LNP-mRNA-based protein replacement/rare disease industry preclinical pipelines.

Drug Candidate	Biological Target	Disease	Company	Website
LUNAR-CF	CFTR ^1^	Cystic Fibrosis	Arcturus	https://arcturusrx.com/pipeline/
LUNAR-CV	undisclosed	rare cardiovascular disease	Arcturus	https://arcturusrx.com/pipeline/
undisclosed	CFTR ^1^	Cystic Fibrosis	Translate Bio	https://translate.bio/pipeline/
undisclosed	undisclosed	Primary Ciliary Dyskinesia	Translate Bio	https://translate.bio/pipeline/
undisclosed	undisclosed	Pulmonary Arterial Hypertension	Translate Bio	https://translate.bio/pipeline/
undisclosed	undisclosed	Idiopathic Pulmonary Fibrosis	Translate Bio	https://translate.bio/pipeline/
undisclosed	undisclosed	Ocular diseases	CureVac	https://www.curevac.com/en/pipeline/
undisclosed	undisclosed	Lung respiratory diseases	CureVac	https://www.curevac.com/en/pipeline/
BNT171	undisclosed	undisclosed	BioNTech/Genevant	https://biontech.de/de/science/pipeline
4 rare disease indications	undisclosed	undisclosed	BioNTech/Genevant	https://biontech.de/de/science/pipeline
mRNA-3283	PAH ^2^	Phenylketonuria	Moderna	https://www.modernatx.com/pipeline
mRNA-3745	G6Pase ^3^	Glycogen Storage Disorder Type 1a	Moderna	https://www.modernatx.com/pipeline
AZD7970	Relaxin-2	Heart Failure	Moderna/AstraZeneca	https://www.modernatx.com/pipeline

^1^ CFTR: cystic fibrosis trans-membrane conductance regulator; ^2^ PAH: phenylalanine hydroxylase; ^3^ G6Pase: glucose-6-phosphatase.

## Data Availability

Not applicable.
